# Application of Bioinspired Structural Ceramics with High-Temperature Electrical Insulation and High Adhesion in K-Type Coaxial Thermocouples

**DOI:** 10.3390/ma18122901

**Published:** 2025-06-19

**Authors:** Zhenyin Hai, Yue Chen, Zhixuan Su, Yemin Wang, Shigui Gong, Yihang Zhang, Shanmin Gao, Chengfei Zhang, Zhangquan Wang, Hongwei Ji, Chenyang Xue, Zhichun Liu

**Affiliations:** 1 School of Aerospace Engineering, Xiamen University, Xiamen 361005, China; haizhenyin@xmu.edu.cn (Z.H.); 35120231151728@stu.xmu.edu.cn (Y.C.); suzhixuan@stu.xmu.edu.cn (Z.S.); wangyemin@stu.xmu.edu.cn (Y.W.); 15259300843@163.com (Y.Z.); gaoshanmin@stu.xmu.edu.cn (S.G.); xuechenyang@xmu.edu.cn (C.X.); 2 School of Optoelectronics and Communications Engineering, Xiamen University of Technology, Xiamen 361005, China; 2322101004@stu.xmut.edu.cn; 3 Inner Mongolia Aerospace Power Machinery Testing Institute, Hohhot 010076, China; zcfdalianfe@126.com (C.Z.); zhangquanw@163.com (Z.W.); 17865320564@163.com (H.J.)

**Keywords:** coaxial thermocouple, silicate, bioinspired, high-temperature insulation, dip-coating

## Abstract

Surface erosion of the coaxial thermocouple probe initiates continuous bridging of thermoelectric materials on the insulation layer surface, forming new temperature measurement junctions. This inherent ability to measure continuous self-erosion ensures the operational reliability of the coaxial thermocouples in high-temperature ablative environments. However, the fabrication of a high-temperature electrical insulation layer and a high-adhesion insulating layer in the coaxial thermocouples remains a challenge. Inspired by calcium carbonate/oxalate crystals in jujube leaves that strengthen the leaves, a bioinspired structural ceramic (BSC) mimicking these needle-like crystals is designed. This BSC demonstrates excellent high-temperature insulation (with insulation impedance of 2.55 kΩ at 1210 °C) and adhesion strength (35.3 Newtons). The BSC is successfully used as the insulating layer in a K-type coaxial thermocouple. The generation rules for surface junctions are systematically studied, revealing that stable and reliable measurement junctions can be created when the sandpaper grit does not exceed 600#. Static test results show that the K-type coaxial thermocouple ranges from 200 °C to 1200 °C with an accuracy of 1.1%, a drift rate better than 0.0137%/h, and hysteresis better than 0.81%. Dynamic test results show that the response time is 1.08 ms. The K-type coaxial thermocouple can withstand a high-temperature flame impact for 300 s at 1200 °C, as well as over forty cycles of high-power laser thermal shock, while maintaining good response characteristics. Therefore, the K-type coaxial thermocouple designed in this study provides an ideal solution for long-term temperature monitoring of the thermal components of aerospace engines under extremely high-temperature, high-speed, and strong thermal shock conditions.

## 1. Introduction

Long-term temperature monitoring of the thermal components of aerospace engines in extreme environments characterized by high temperatures, high speeds, and intense thermal shock has always been a challenge [1,2]. In high-temperature ablation environments, thermal components often face extreme conditions such as intense thermal radiation, airflow impact, and high-temperature ablation [3]. Therefore, stable and reliable temperature monitoring technology remains a significant challenge in the aerospace field. Existing temperature sensors, such as thin-film thermocouples [4], thin-film thermistors [5,6,7], and fiber-optic temperature sensors [8,9], are limited by factors such as thermal shock resistance, high-temperature performance, vibration resistance, environmental media, and installation conditions. As a result, they cannot meet the practical application requirements.

Coaxial thermocouples, as a type of continuous self-eroding sensor, have gradually shown potential for use in high-temperature ablation environments. Their unique structure provides advantages such as fast response, reusability, and adaptability to extreme environments, which makes them promising for aerospace applications [10]. However, the fabrication of a high-temperature electrical insulation layer and a high-adhesion insulating layer in K-type coaxial thermocouples remains a challenge. Currently, most studies use epoxy resin as the electrical insulating layer in coaxial thermocouples, aiming to measure transient temperature or transient heat flux density [11,12,13]. Byrenn Birch et al. have used alumina as the electrical insulating layer to fabricate E-type coaxial thermocouples, opening up possibilities beyond epoxy resin as the insulating material [14]. Jun Chen et al. have used high-temperature adhesive as the electrical insulating layer to fabricate tungsten–rhenium coaxial thermocouples for ultra-high-temperature measurements [15]. The high-temperature adhesive also achieves reliable connection of the positive and negative electrode materials. Therefore, the improvement of the insulation performance of coaxial thermocouples is the key to realizing long-term high-temperature measurements.

The structure of a coaxial thermocouple consists of three main components: a metal wire, a metal tube, and an insulating layer. The metal tube and wire are made of different materials, with the insulating layer serving to electrically isolate the wire from the tube. However, the fabrication of high-temperature electrically insulating ceramic layers for high-curvature metal wires remains one of the primary challenges. Traditional ceramic materials often face issues such as cracking, delamination, and poor adhesion when applied to metal wires, which limits their performance in high-temperature environments [16]. Although progress has been made by exploring new ceramic materials and improving fabrication processes, the key challenge remains in developing ceramic insulating layers with high-temperature resistance, high insulation, and strong adhesion on high-curvature metal wires.

This paper reports on the application of BSC with high-temperature electrical insulation and high adhesion in K-type coaxial thermocouples, addressing the challenge of fabricating high-temperature BSC electrical insulating layers on high-curvature metal wires. Inspired by calcium carbonate/oxalate needle crystals in jujube leaves that boost leaf strength, a bioinspired ceramic mimics these crystals. This BSC exhibits excellent high-temperature electrical insulation and high adhesion. The K-type coaxial thermocouple is fabricated with BSC as the electrical insulating layer. The static and dynamic performance of the K-type coaxial thermocouple is comprehensively evaluated. Finally, the engineering application capabilities of the K-type coaxial thermocouple are assessed under simulated extreme conditions. This work provides an ideal solution for long-term temperature monitoring of the thermal components of aerospace engines under extremely high-temperature, high-speed, and strong thermal shock conditions.

## 2. Materials and Methods

### 2.1. Materials

Ni-Cr10 tubes (outer diameter 2 mm, inner diameter 0.52 mm, length 20 mm), Ni-Cr10 wires (diameter 0.5 mm), and Ni-Si3 wires (outer diameter 0.5 mm) are produced by Yuguanghong Trading Co., Ltd., Zhouzhen Town, Kunshan City, China. The silicate composite material used to prepare the high-temperature electrical insulating layer is purchased from Pfizer Electronics Co., Ltd., Dongguan, China. The silicate composite material mainly consists of CaO, Al_2_O_3_, and SiO_2_. A B-type thermocouple is produced by Xinghua Shunsheng Electric Co., Ltd. (Hefei, China), with a tolerance of ±1.5 °C.

### 2.2. Processing, Testing and Characterization Equipment

The dip-coating machine (DR-TP600, Guangdong South China Technology Science and Technology Co., Ltd., Guangzhou, China) is used to coat the insulating layer on the surface of the Ni-Si3 wire. The laser welding machine (AHL-W600III, Shenzhen Aohua Laser Technology Co., Ltd., Shenzhen, China) is used for welding the Ni-Cr10 wire to the Ni-Cr10 tube. The tube furnace (OTF-1200X, Hefei Kejing Material Technology Co., Ltd., Hefei, China) is utilized for sintering and performance testing of the CT. A digital multimeter (DAQ6510, TEKTRONIX, INC., Beaverton, OR, USA) and the rugged miniature dynamic data acquisition and analysis system (DH5916, Jiangsu Donghua Testing Technology Co., Ltd., Taizhou, China) measure the thermoelectric potential of the CT. A scanning electron microscope (SUPRA55 SAPPHIRE, Carl Zeiss AG, Oberkochen, Germany) captures scanning electron microscope images. The metallographic microscope (MV3000, Jiangnan Yongxin Optical Co., Ltd., Nanjing, China) is used to observe the microstructure of the friction welding joints.

### 2.3. Preparation of K-Type Coaxial Thermocouple

**Figure 1:**
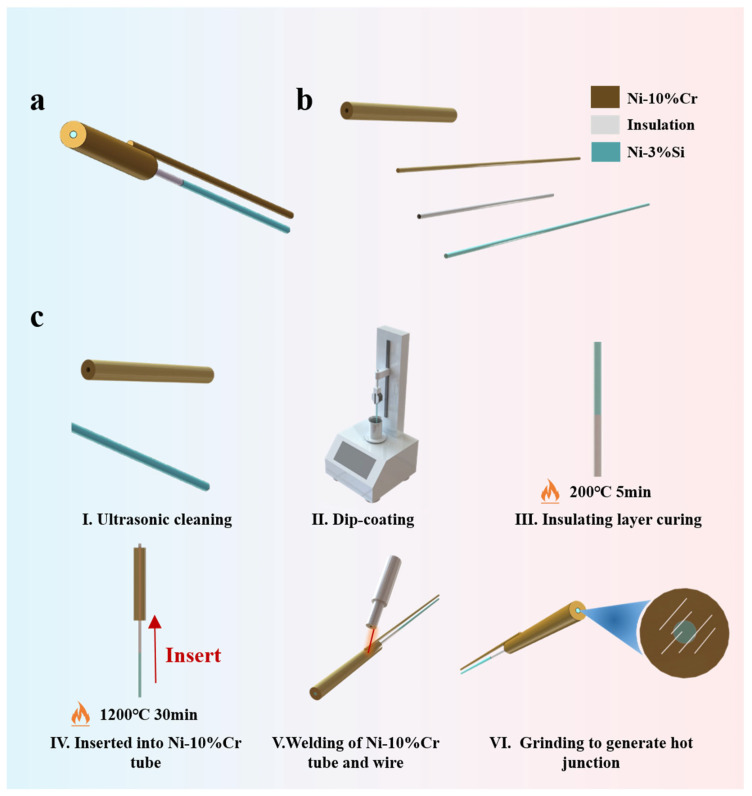
(**a**) Structure of the K-type coaxial thermocouple; (**b**) exploded view of the K-type coaxial thermocouple; (**c**) fabrication process of the K-type coaxial thermocouple.

The structure of the K-type coaxial thermocouple consists of four parts: a Ni-Cr10 tube, a Ni-Cr10 wire, a Ni-Si3 wire, and an electrical insulating layer. Figure 1a shows the structure of the K-type coaxial thermocouple, while Figure 1b shows an exploded view of the K-type coaxial thermocouple. A silicate composite ceramic, known for its low thermal expansion coefficient, excellent high-temperature performance, and dielectric properties, is chosen as the electrical insulating layer [17]. The dip-coating method, which is well-established, is employed for coating, as it is cost-effective and suitable for curved surface coating [18,19].

**Figure 2:**
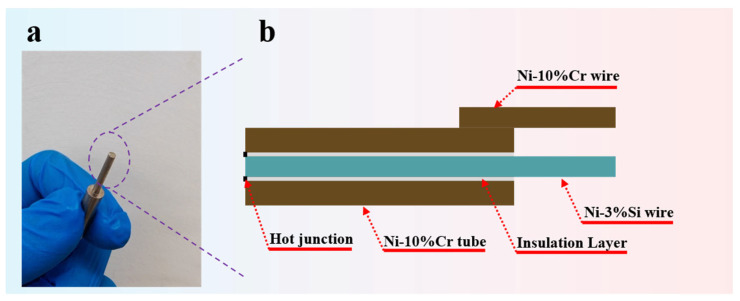
(**a**) Physical image of the K-type coaxial thermocouple; (**b**) detailed cross-sectional structure of the K-type coaxial thermocouple.

To elaborate on the manufacturing technique of the K-type coaxial thermocouple, the fabrication process is shown in Figure 1c. The first step involves using ultrasonic cleaning technology to pretreat the Ni-Cr10 tube and Ni-Si3 wire in acetone, deionized water, and alcohol to remove impurities and oxides from their surfaces. The second step involves using the dip-coating method to prepare the BSC electrical insulating layer on the Ni-Si3 wire. The dip time is 120 s, the drawing speed is 20 μm/s, and the dip depth is 40 mm. The third step involves placing the Ni-Si3 wire with BSC electrical insulating layer in a drying oven at 200 °C for 5 min. The fourth step involves inserting the Ni-Si3 wire with the BSC electrical insulating layer into a Ni-Cr10 tube. The assembly is then placed in a high-temperature tube furnace to sinter the BSC electrical insulating layer, with the sintering temperature set at 1200 °C for 30 min. The fifth step involves welding the Ni-Cr10 tube to the Ni-Cr10 wire. Finally, sandpaper is used to rub the surface of the temperature measurement probe, forming the temperature measurement junction. Figure 2a shows an image of the K-type coaxial thermocouple, while Figure 2b provides detailed information on the cross-sectional structure of the K-type coaxial thermocouple.

### 2.4. Performance Parameter Evaluation Method

The primary performance parameters of the thermocouple are hysteresis, stability/drift, accuracy, and response time. Hysteresis refers to the offset between the forward temperature excitation and the reverse temperature excitation at the thermocouple output value at a given temperature. Hysteresis is calculated by the following formula:

(1) $$\mathrm{Hysteresis}=\frac{{\Delta T|}_{V=x}}{T_{F.S.}}\times 100\%$$

where ${\Delta T|}_{V=x}$ denotes the deviation ΔT between the forward and reverse temperature excitations at the thermocouple output voltage value, and $T_{F.S.}$ denotes the calibration temperature range.

Stability/drift represents the ability of the thermocouple to maintain its performance parameters over a certain period of time. Stability/drift is calculated as follows:

(2) $$\mathrm{Drift}/\mathrm{Stability}=\frac{{T_{\mathrm{CT}}|}_{\mathrm{Temp}=x,\mathrm{Time}=y}-{T_{B}|}_{\mathrm{Temp}=x,\mathrm{Time}=y}}{T_{F.S}}\times 100\%$$

where ${T_{\mathrm{CT}}|}_{\mathrm{Temp}=x,\mathrm{Time}=y}$ denotes the output temperature value of the K-type coaxial thermocouple at a temperature of x °C and a holding time of y hours. ${T_{B}|}_{\mathrm{Temp}=x,\mathrm{Time}=y}$ denotes the output temperature value of a standard B-type thermocouple at a temperature of x ℃ and a holding time of y hours. $T_{F.S}$ denotes the full-scale temperature.

Accuracy is calculated by the following formula:

(3) $$\mathrm{Accuracy}=\frac{\Delta T}{T_{F.S.}}\times 100\%$$

where ΔT denotes the temperature deviation between the thermocouple being measured and the standard thermocouple, and $T_{F.S.}$ denotes the calibration temperature range.

The response time ($\tau_{0.632}$) is calculated using the following formula:

(4) $$\tau_{0.632}=\Delta t\times 63.2\%$$

where Δt denotes the time for the thermocouple to reach a steady state after being excited by a heat source.

The applicable temperature range of the performance parameters calculated by the above formula is 200 °C to 1200 °C.

## 3. Results and Discussion

**Figure 3:**
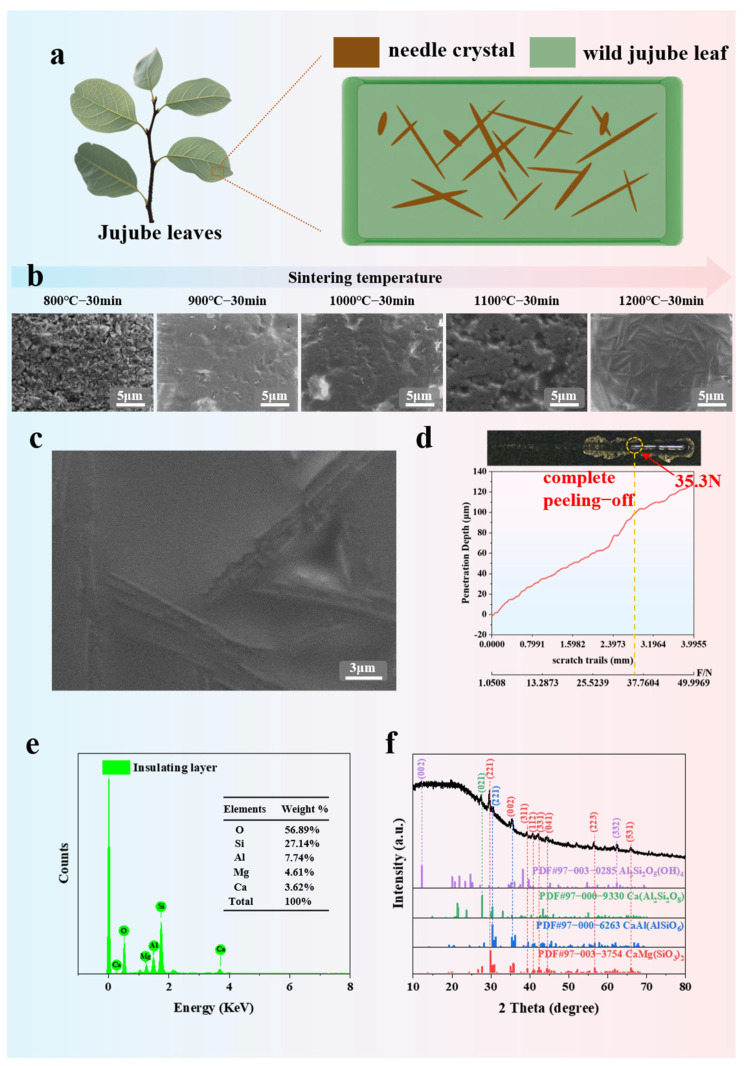
(**a**) Schematic of calcium carbonate/oxalate needle crystals secreted by jujube leaves; (**b**) microstructural evolution of the BSC at different sintering temperatures; (**c**) close-up of the BSC microstructure; (**d**) adhesion strength test results of the BSC; (**e**) EDS spectra; (**f**) XRD patterns.

To ensure the reliable application of the K-type coaxial thermocouple, it is crucial that the electrical insulating layer material can achieve reliable adhesion with the Ni-Cr10 tube and Ni-Si3 wire. Previous studies have reported that jujube leaves, which grow in extreme environments such as arid and sandy conditions, are more likely to secrete calcium carbonate/oxalate needle crystals (as shown in Figure 3a), and the presence of these needle crystals helps to enhance the mechanical strength of the leaves [20]. Inspired by this, a bioinspired ceramic mimicking jujube leaf crystals is designed. This BSC demonstrates excellent high-temperature electrical insulation and strong adhesion.

Figure 3b shows the microstructural evolution of BSC at different sintering temperatures. Research reveals that the BSC begins to crystallize and form a glass phase at 900 °C, consistent with previous reports [21]. When the temperature reaches 1200 °C, needle-like crystals begin to precipitate [22]. Figure 3c provides more details of the needle-like crystals, and the SEM characterization results confirm that these needle-like crystals are very similar to the calcium carbonate/oxalate crystals secreted by jujube leaves.

To further verify the adhesion properties of these needle-like crystals, a scratch test is conducted to evaluate the adhesion strength between the electrical insulating layer and the metal substrate. For the test, a nickel-based substrate (volume: 20 × 30 × 2 mm^3^) is chosen because its thermal-physical properties are similar to those of the Ni-Cr10 and Ni-Si3 alloys [23,24]. As shown in Figure 3d, the scratch length is 3.9955 mm, and the vertical load on the indenter gradually increases from 1.0508 N to 49.9969 N during the scratching process. When the sample reaches a critical load of 35.3 N, the film completely peels off, exposing large continuous metallic scratches on the nickel-based substrate surface. Since the loading is gradual, it can be assumed that the complete peeling-off load for the film is 35.3 N. The reported adhesion strength of titanium–aluminum coatings on Si_4_N_4_ ceramic tools (33.7 Newtons) [25] demonstrates extremely high adhesion strength, providing data to prove that the electrical insulating layer reliably adheres to both the Ni-Cr10 tube and the Ni-Si3 wire.

Further detailed analysis of the elemental composition, material constitution, and phase structure of these needle-like crystals was conducted. As presented in Figure 3e, the EDS spectrum of the needle-like crystals reveals weight percentages of O (56.89%), Si (27.14%), Al (7.74%), Mg (4.61%), and Ca (3.62%). Figure 3f displays the XRD diffraction pattern, wherein diffraction peaks at 12.1° and 62.4° correspond to characteristic peaks of Al_2_Si_2_O_5_(OH)_4_; the peak at 27.4° matches Ca(Al_2_Si_2_O_8_); the peak at 30.3° aligns with CaAl(AlSiO_6_); and peaks at 29.6°, 35.3°, 39.2°, 40.7°, 42.1°, 44.4°, 56.4°, and 66° are assigned to CaMg(SiO_3_)_2_. These results collectively confirm that the needle-like crystals consist of four distinct substances: Al_2_Si_2_O_5_(OH)_4_, Ca(Al_2_Si_2_O_8_), CaAl(AlSiO_6_), and CaMg(SiO_3_)_2_.

The temperature measurement junction of the coaxial thermocouple, also known as the friction welding junction, is an effective method for connecting dissimilar metals [26]. This type of friction-welded temperature measurement junction has the advantage of a small volume, which allows the coaxial thermocouple to have fast response characteristics [27]. The formation of the friction-welded junction is influenced by the plastic deformation of the metal materials and the cracks in the electrical insulating layer. Specifically, the greater the plastic deformation ability of the metal materials, the easier it is to form the temperature measurement junction. The more voids in the electrical insulating layer, the more likely a temperature measurement junction will form inside the sensor, leading to a decrease in measurement accuracy. Therefore, analyzing the formation of the friction-welded measurement junction and the surface quality of the coaxial thermocouple is of great importance.

**Figure 4:**
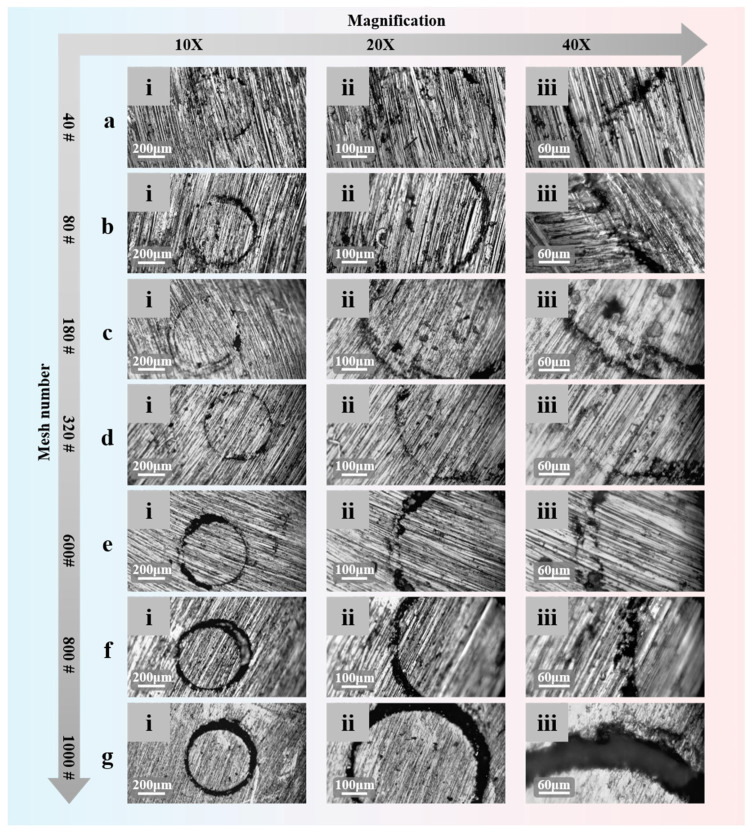
Microstructure of the temperature-sensing surface of the K-type coaxial thermocouple after polishing with sandpapers of different grits: (**a**) 40# grit sandpaper polishing; (**b**) 80# grit sandpaper polishing; (**c**) 180# grit sandpaper polishing; (**d**) 320# grit sandpaper polishing; (**e**) 600# grit sandpaper polishing; (**f**) 800# grit sandpaper polishing; (**g**) 1000# grit sandpaper polishing; (**i**) magnifications of 10×; (**ii**) magnifications of 20×; (**iii**) magnifications of 40×.

Figure 4 shows the microscopic images of the temperature-sensing surface of the K-type coaxial thermocouple after polishing with sandpapers of grit sizes 40#, 80#, 180#, 320#, 600#, 800#, and 1000# at magnifications of 10X, 20X, and 40X. When the sandpaper grit size does not exceed 600#, stable and reliable measurement junctions can be formed, which is crucial for its engineering application. Figure 4g(i–iii) show the microscopic images of the electrical insulating layer after polishing with 1000# sandpaper. The black areas represent the BSC electrical insulating layer, and the images show that no cracks have appeared on the surface of the BSC electrical insulating layer after polishing with different grit sizes, indicating that the BSC electrical insulating layer has good toughness.

**Figure 5:**
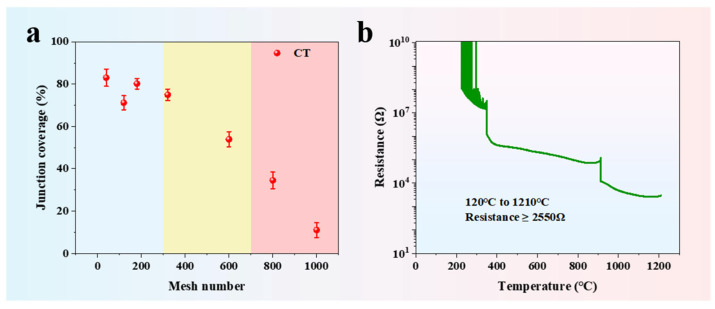
(**a**) Node coverage rate of the temperature-sensing surface of the coaxial thermocouple; (**b**) insulation performance test results of the BSC electrical insulating layer.

To quantitatively describe the impact of different sandpaper grit sizes on the formation of friction measurement junctions, the ImageJ software (version number: v0.5.8) is used to statistically analyze the node coverage of the measurement junctions. For the measurement junctions prepared using seven grit sizes of sandpaper (40#, 80#, 180#, 320#, 600#, 800#, and 1000#), three parallel samples were tested for each grit size. As shown in Figure 5a, the mean node coverage rates and standard deviations were as follows: 40# (83.05% ± 4.03%), 80# (71.22% ± 3.31%), 180# (80.18% ± 2.46%), 320# (74.98% ± 2.69%), 600# (53.91% ± 3.56%), 800# (34.54% ± 3.93%), and 1000# (11.07% ± 3.48%). This also confirms that reliable measurement junctions are more likely to form when the sandpaper grit does not exceed 600#. The insulation performance of the BSC electrical insulating layer is directly related to the measurement accuracy of the coaxial thermocouple. At 1210 °C, the resistance of the BSC electrical insulating layer is as high as 2.55 kΩ, ensuring the accuracy of temperature measurements in high-temperature environments.

**Figure 6:**
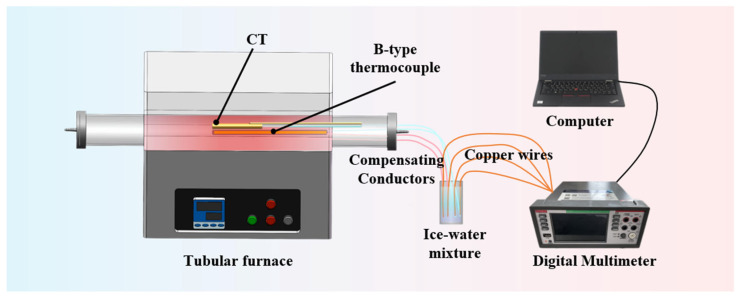
Static calibration testing platform for the K-type coaxial thermocouple.

In order to ensure the reliable application of the coaxial thermocouple, static calibration tests are conducted. Figure 6 shows the static calibration testing platform for the K-type coaxial thermocouple, which mainly consists of a tube furnace, an ice–water mixture, a digital multimeter, a B-type thermocouple, and a computer. The hot ends of the B-type thermocouple and the K-type coaxial thermocouple are placed in the uniform temperature zone of the tube furnace to ensure both types of thermocouples are exposed to the same temperature environment during testing. The cold ends are placed in an ice–water mixture through compensation wires to eliminate cold-end errors in the thermocouple readings. The thermocouples are connected to a data acquisition system through copper wires, and the collected data are stored in a computer. The target temperature is controlled by the temperature control program of the tube furnace.

**Figure 7:**
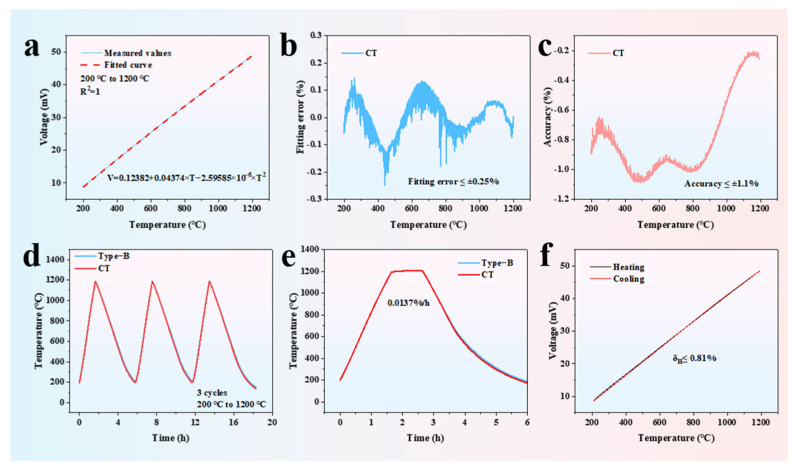
(**a**) Fitting curve of the K-type coaxial thermocouple; (**b**) fitting error curve of the K-type coaxial thermocouple; (**c**) accuracy curve of the K-type coaxial thermocouple; (**d**) three-cycle heating and cooling test results; (**e**) drift rate test results of the K-type coaxial thermocouple; (**f**) hysteresis test results of the K-type coaxial thermocouple.

Currently, there have been many reports on K-type coaxial thermocouples, but there are almost no studies on K-type coaxial thermocouples that can be used for long-term high-temperature monitoring. Therefore, it is necessary to perform static calibration tests on the K-type coaxial thermocouple designed and fabricated in this study. The voltage curves of the K-type coaxial thermocouple are fitted twice in the temperature range from 200 °C to 1200 °C, with an R^2^ value of 1 (as shown in Figure 7a), and the fitting error is less than 0.25% (as shown in Figure 7b). To evaluate the accuracy of the K-type coaxial thermocouple, the temperature error between the K-type coaxial thermocouple and the standard B-type thermocouple is compared. The test results show that the K-type coaxial thermocouple has excellent accuracy, with an accuracy of 1.1% (as shown in Figure 7c). Figure 7d shows the three-cycle heating and cooling test curve, indicating that the K-type coaxial thermocouple can operate continuously for over 18 h, demonstrating its ability to work in continuous high- and low-temperature environments. The drift rate test curve for the K-type coaxial thermocouple is shown in Figure 7e, and the test results indicate that the K-type coaxial thermocouple has excellent stability, with a drift rate of 0.0137%/h. Figure 7f shows the heating and cooling test curve for the K-type coaxial thermocouple, and the test results show that the hysteresis is better than 0.81%. Therefore, the static calibration test results demonstrate that the K-type coaxial thermocouple exhibits excellent performance. The comparison between the K-type coaxial thermocouple based on BSC and other types of temperature sensors in terms of accuracy, drift rate, response time and temperature range is shown in Table 1.

**Figure 8:**
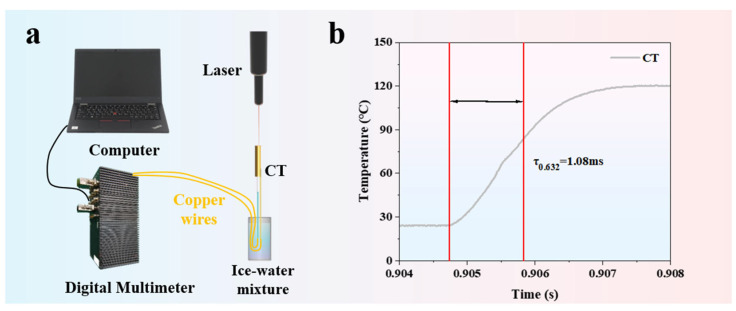
(**a**) Dynamic calibration testing platform for the K-type coaxial thermocouple; (**b**) response time test results of the K-type coaxial thermocouple.

The dynamic response characteristics reflect the sensor’s ability to react to changes in external excitation. Figure 8a shows the dynamic calibration testing platform for the K-type coaxial thermocouple, which mainly consists of a digital multimeter, a computer, an ice–water mixture, and a laser. The response time of the K-type coaxial thermocouple is 1.08 ms (as shown in Figure 8b), indicating that it has fast response capability.

**Figure 9:**
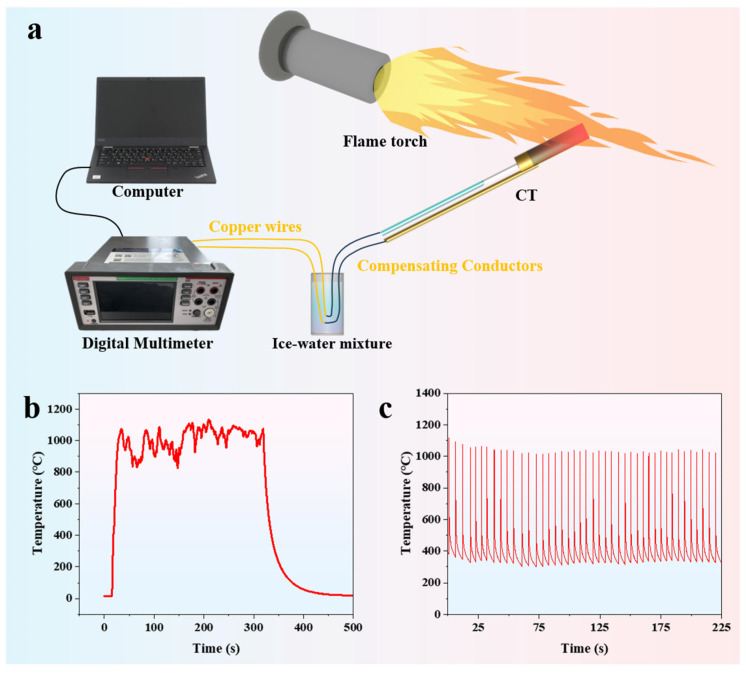
(**a**) High-temperature flame impact testing platform; (**b**) flame impact test results; (**c**) high-power laser cyclic thermal shock test results.

To further demonstrate the practical application capabilities of the K-type coaxial thermocouple in extreme environments, high-temperature flame impact and high-power laser thermal shock tests are conducted. In the high-temperature flame impact test, the high-temperature flame generated by a flame spray gun is directed at the temperature-sensing probe of the K-type coaxial thermocouple (as shown in Figure 9a). The K-type coaxial thermocouple quickly responds and endures the thermal shock of the flame for up to 300 s. Due to the shaking of the flame spray gun held by the experimenter and convective heat exchange in the air, the temperature curve fluctuates significantly (as shown in Figure 9b). As shown in Figure 9c, after enduring over forty cycles of high-power laser thermal shock, the K-type coaxial thermocouple still maintains good response characteristics. These experimental results demonstrate that the K-type coaxial thermocouple possesses the capability to function effectively in extreme environmental engineering applications.

## 4. Conclusions

Inspired by the natural mechanism of calcium carbonate/oxalate needle crystals strengthening jujube leaves, this study successfully designed the BSC featuring a needle-like crystal structure. The BSC exhibits excellent high-temperature electrical insulation (with an insulation impedance of 2.55 kΩ at 1210 °C) and strong adhesion (35.3 Newtons), and has been effectively integrated as the electrical insulating layer in the K-type coaxial thermocouple. Systematic investigation of surface junction generation rules established the critical process criterion that stable and reliable measurement junctions form when the sandpaper grit does not exceed 600#. Static tests confirmed that the thermocouple achieves an accuracy of 1.1% within 200 °C to 1200 °C, a drift rate better than 0.0137%/h at 1200 °C, and hysteresis better than 0.81%. Dynamic tests verified a response time of 1.08 ms. The K-type coaxial thermocouple maintained fast response characteristics under extreme conditions, including 1200 °C high-temperature flame shock for 300 s and over forty cycles of high-power laser thermal shock. The K-type coaxial thermocouple with the BSC electrical insulating layer developed in this work provides an ideal solution for long-term temperature monitoring of hot components in aerospace engines under extreme high-temperature, high-speed, and strong thermal shock conditions.

## Figures and Tables

**Table 1:** Comparison of K-type coaxial thermocouple based on BSC with other types of temperature sensors.

Sensor Type	Temperature Test Range	Accuracy	Drift Rate	Response Time	Reference
ITO/In_2_O_3_ thin-film thermocouple	0 °C–1275 °C	\	8.34%/h	\	[28]
W/W-26Re thin-film thermocouple	100 °C–1600 °C	1.5%	0.26%/h	1.74 s	[29]
Polymer-derived ceramic composite thin-film thermistor	100 °C–1100 °C	\	1.2%/h	\	[5]
K-type coaxial thermocouple	200 °C–1200 °C	1.1%	0.0137%/h	1.08 ms	This work

## Data Availability

The raw data supporting the conclusions of this article will be made available by the authors on request.
